# Conversion of Polypropylene Waste into Value-Added Products: A Greener Approach

**DOI:** 10.3390/molecules27093015

**Published:** 2022-05-07

**Authors:** Jan Nisar, Maria Aziz, Afzal Shah, Iltaf Shah, Munawar Iqbal

**Affiliations:** 1 National Centre of Excellence in Physical Chemistry, University of Peshawar, Peshawar 25120, Pakistan; azizmaria544@gmail.com; 2 Department of Chemistry, Quaid-i-Azam University, Islamabad 45320, Pakistan; 3 Department of Chemistry, College of Science, United Arab Emirates University, Al Ain P.O. Box 15551, United Arab Emirates; 4 Department of Chemistry, Division of Science and Technology, University of Education, Lahore 54000, Pakistan; bosalvee@yahoo.com

**Keywords:** plastic waste, pyrolysis, oil, fuel properties, kinetic parameters, waste management

## Abstract

Plastic has made our lives comfortable as a result of its widespread use in today’s world due to its low cost, longevity, adaptability, light weight and hardness; however, at the same time, it has made our lives miserable due to its non-biodegradable nature, which has resulted in environmental pollution. Therefore, the focus of this research work was on an environmentally friendly process. This research work investigated the decomposition of polypropylene waste using florisil as the catalyst in a salt bath over a temperature range of 350–430 °C. A maximum oil yield of 57.41% was recovered at 410 °C and a 40 min reaction time. The oil collected from the decomposition of polypropylene waste was examined using gas chromatography-mass spectrometry (GC-MS). The kinetic parameters of the reaction process were calculated from thermogravimetric data at temperature program rates of 3, 12, 20 and 30 °C·min^−1^ using the Ozawa–Flynn–Wall (OFW) and Kissinger–Akahira–Sunnose (KAS) equations. The activation energy (Ea) and pre-exponential factor (A) for the thermo-catalytic degradation of polypropylene waste were observed in the range of 102.74–173.08 kJ·mol^−1^ and 7.1 × 10^8^–9.3 × 10^11^ min^−1^ for the OFW method and 99.77–166.28 kJ·mol^−1^ and 1.1 × 10^8^–5.3 × 10^11^ min^−1^ for the KAS method at a percent conversion (α) of 0.1 to 0.9, respectively. Moreover, the fuel properties of the oil were assessed and matched with the ASTM values of diesel, gasoline and kerosene oil. The oil was found to have a close resemblance to the commercial fuel. Therefore, it was concluded that utilizing florisil as the catalyst for the decomposition of waste polypropylene not only lowered the activation energy of the pyrolysis reaction but also upgraded the quantity and quality of the oil.

## 1. Introduction

In recent decades, a tremendous growth in plastic utilization has occurred as a result of the rising population and people’s desire to improve their living standard [1]. Plastics have been widely used in everyday life since their discovery, owing to their appealing properties such as their light weight, stiffness, durability and competitive prices. Due to their properties such as their toughness and durability, plastics are very helpful in making the life of people comfortable; however, at the same time, they also pose serious environmental issues due to resistance to natural breakdown and create major obstacles to waste management [2]. Therefore, the quantity of plastic waste is increasing every day all over the world, resulting in serious environmental pollution [3,4].

If plastic consumption continues with its current flow, then worldwide plastic waste will jump from 260 million tons/year, as estimated in 2016, to 460 million tons every year until 2030 [5]. For the handling of discarded plastic, various strategies are in use: e.g., the concept of the 3Rs, which stands for reuse, reduce and recycle, is a basic and widely used waste management policy [6]. Some traditional waste recycling procedures are termed “mechanical recycling” that consists of primary and secondary recycling. Primary recycling is the fundamental recovery of uncontaminated plastic objects and particles for re-use in related applications. It has the disadvantage of having a limited number of re-use cycles and being confined to industrial materials with low contamination [7]. Meanwhile, secondary recycling includes sorting, grinding, washing and extrusion, where the resultant component might possibly be indistinguishable from the original. However, these methods only recycle 15 to 20% of total plastic waste [8].

Other methods for disposing of and recycling plastic waste are landfilling, incineration and chemical recycling. Incineration is the process of burning plastic waste in open areas. Incineration is becoming a severe issue and endangering both human health and the environment as it produces a wide range of volatile and gaseous contaminants that can damage the environmental conditions. Incineration has also been admonished for producing large volumes of bottom ash as well as a variety of harmful air pollutants such as polycyclic aromatic hydrocarbons and dioxins in the case of halogen-containing polymers [9].

Landfilling, or land disposal, is the most widely used traditional method. This is the most popular and convenient method of waste disposal. However, the increased filling of plastic waste into landfills has resulted in pollution of the soil, water and air. Moreover, the public’s reliance on landfilling has resulted in environmental, health and safety issues. Because of the high costs and the poor degradability of plastic-based products, landfills are becoming an unappealing method of disposal [10].

Chemical recycling refers to the thermal depolymerization of plastic waste into valuable products. Sometimes, simple thermal decomposition does not produce the desired results [11]; therefore, the thermo-catalytic method is used to convert plastic waste into useful products [12]. Catalytic pyrolysis is beneficial over thermal pyrolysis in several aspects, including the lower degradation temperature, quicker cracking reaction, improved selectivity, shorter operating time and enhanced product yields [13]. Therefore, pyrolysis can rightly be termed as a greener approach because it results in the disposal of plastics along with the production of some value-added products, whereas other methods such as incineration and landfilling lead to contamination of the soil, water and air.

Polypropylene is a low-cost thermoplastic polymer which makes up 26% of the worldwide polymer market. Polypropylene has a lower density and a higher service temperature and is harder and more rigid, with high resistance to environmental stress cracking, and it is more vulnerable to oxidation and chemical assault. Pyrolysis of pure and waste polypropylene has been studied in different reactors using various catalysts [14,15,16,17,18]; however, no attention has been paid to the use of florisil as the catalyst for the decomposition of waste polypropylene in a salt bath reactor. Therefore, the objectives of this research were to devise an appropriate technique for the degradation of polypropylene waste using florisil as the catalyst and to study the kinetics of the pyrolysis reaction. This study will help in the degradation of polypropylene waste on a pilot scale.

## 2. Results and Discussion

### 2.1. Thermogravimetric Analysis

**Figure 1:**
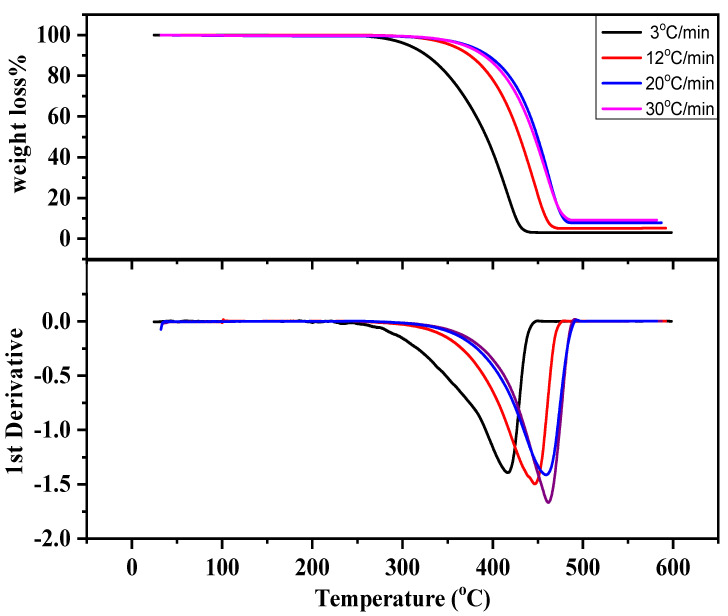
TG/DTG curves of PP waste obtained at various temperature program rates.

Thermogravimetric analysis of waste PP was conducted in a nitrogen atmosphere at temperature program rates of 3, 12, 20 and 30 °C·min^−1^ in the presence of florisil. The weight loss curves of polypropylene waste at various temperature program rates are depicted in Figure 1. The figure shows that with the increase in the heating rate, the degradation curves shift to a high temperature for the same degree of conversion due to the heat transfer lag. The results agree well with our previous investigations [19]. Moreover, the DTG curves were observed to have a single depth which depicts polypropylene degradation as a result of breakage of the polymer chain in a single step. Li et al. [20] studied the thermal degradation of PP at a temperature ranging from 50 to 750 °C at 5 °C·min^−1^ and observed that the maximum degradation of PP occurred at 429 °C with a single-step degradation.

**Figure 2:**
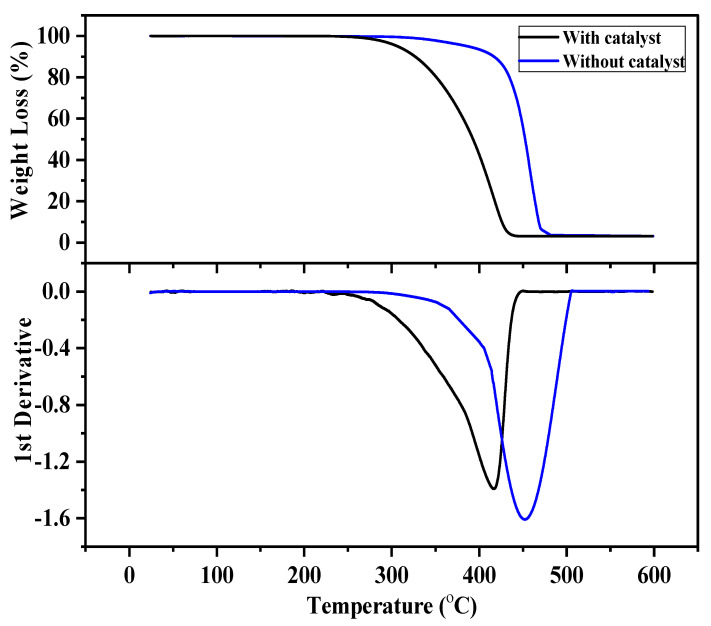
TG/DTG of waste PP with and without florisil at 3 °C·min^−1^.

Figure 2 demonstrates the effect of the florisil catalyst on the degradation of waste polypropylene. It can be easily seen from the figure that without the catalyst, polypropylene degradation initiated at 285 °C and came to an end at 480 °C, while in the presence of the catalyst, it started at 222 °C and finished at 438 °C. The TG/DTG curves show that waste polypropylene without the catalyst completely degraded at about 500 °C, while in the presence of the florisil catalyst, it degraded at about 450 °C. The results are in consonance with reported studies. As observed by Tekin et al. [21] waste PP completely decomposed at 500 °C, and significant weight loss steps occurred in the temperature range of 340–485 °C. Obali et al. [22] analyzed polypropylene degradation thermogravimetrically as well as in the presence of an alumina-loaded mesoporous catalyst and revealed that virgin polypropylene degraded between 350 and 480 °C, while with the catalyst, the degradation temperature reduced to 253–360 °C.

### 2.2. Kinetic Study

Waste polypropylene with and without the catalyst was decomposed in a thermogravimetric analyzer at temperature program rates of 3, 12, 20 and 30 °C·min^−1^, and the resultant data were utilized for determining the kinetic parameters applying the OFW and KAS methods.

#### 2.2.1. Ozawa–Flynn–Wall Equation

**Figure 3:**
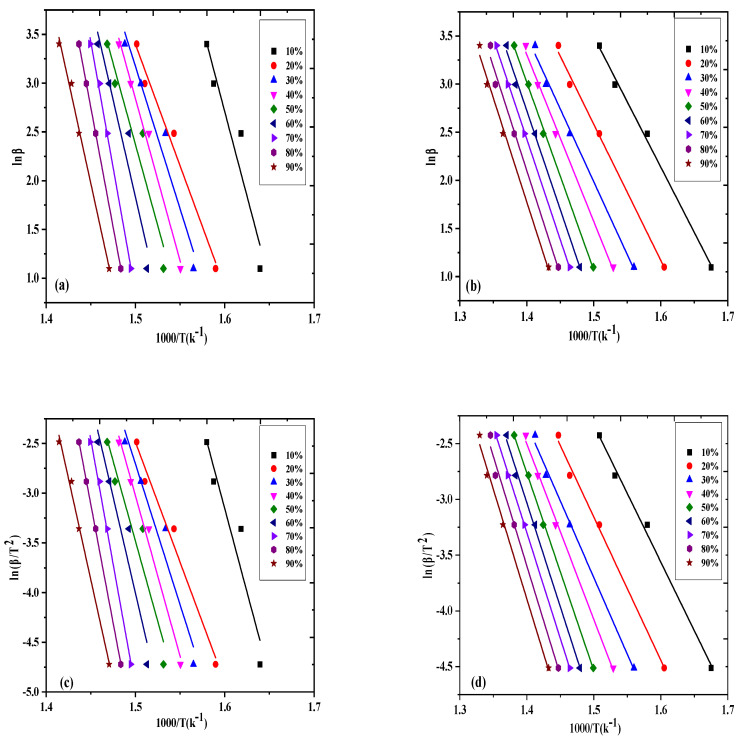
OFW plot for degradation of waste PP (**a**) without the catalyst and (**b**) with the catalyst at various conversions. KAS plot for degradation of waste PP (**c**) without the catalyst and (**d**) with the catalyst at various conversions.

The correlation between lnβ and 1/T plotted using Equation (1) at various percent conversions for the virgin sample is depicted in Figure 3a and for the catalyzed reaction in Figure 3b. The activation energy and A-factor determined from the slope and intercept of the plots are presented in Table 1. The Ea and A for the uncatalyzed reaction ranged from 109.95 to 198.66 kJ·mol^−1^ and 9.1 × 10^8^ to 9.3 × 10^12^ min^−1^, respectively. The data obtained are in agreement with some earlier studies. Briceno et al. [23] calculated the Ea for PP degradation in the range of 177–194 kJ·mol^−1^ using the OFW method, with conversion ranging from 10 to 90% at different heating rates. Wu et al. [24] found an Ea of 183.6 kJ·mol^−1^ for commercial-grade polypropylene degradation using the Friedman method.

For the catalyzed reaction, the Ea values were also observed to increase with the percent conversion; however, the activation energy values obtained were lower compared to those of the uncatalyzed reaction, i.e., the Ea values ranged from 102.74 to 173.08 kJ·mol^−1^ and the pre-exponential factor was observed to range from 7.1 × 10^8^ to 9.3 × 10^11^ min^−1^ with the increase in percent conversion from 0.1 to 0.9. Almost similar results were also obtained by Das and Tiwari using the OFW equation [25]. Lin et al. [26] studied polypropylene thermo-catalytic degradation and observed a reduction in Ea (143.4 kJ·mol^−1^) using a silicalite catalyst as compared to without the catalyst (147.5 kJ·mol^−1^), using the Ozawa method.

#### 2.2.2. Kissinger–Akahira–Sunnose Equation

Figure 3c presents the plot of ln(β/T^2^) against 1/T at various degrees of conversion for virgin waste propylene using Equation (2). From the plots, Ea and the A-factor were calculated as listed in Table 1. Ea was noted to increase from 108.76 to 184.28 kJ·mol^−1^ with the increase in percent conversion from 0.1 to 0.9. Briceno et al. [23] calculated the Ea for the thermal degradation of polypropylene using the Kissinger–Akahira–Sunnose method and found Ea values of 174–191 kJ·mol^−1^. Aboulkas et al. [27] utilized the Kissinger–Akahira–Sunnose approach to calculate the activation energy for various conversions for PP degradation. The average activation energy computed for PP was 179 kJ·mol^−1^. In another study, municipal plastic waste including polypropylene was analyzed thermogravimetrically at 30–700 °C. The activation energy for polypropylene degradation was calculated as 261.22 kJ·mol^−1^ using the KAS equation [28]. Similarly, for catalyzed PP degradation, the plot of ln(β/T^2^) against 1/T was constructed according to Equation (2), as depicted in Figure 3d, and the kinetic parameters calculated from the plots are presented in Table 1. The table shows that as the percent conversion increased, Ea also increased from 99.77 to 166.28 kJ·mol^−1^. The rise in these values was mainly attributed to polypropylene complex reactions, involving multiple reaction steps. Esmizdeh et al. [29] revealed an increase in Ea with conversion and reported variation in the activation energy from 70 to 160 kJ·mol^−1^. Das and Tiwari [25] examined the thermal degradation of four plastics and observed an increase in the activation energy (136–173 kJ·mol^−1^) with the percent conversion.

### 2.3. Pyrolysis of Polypropylene

**Figure 4:**
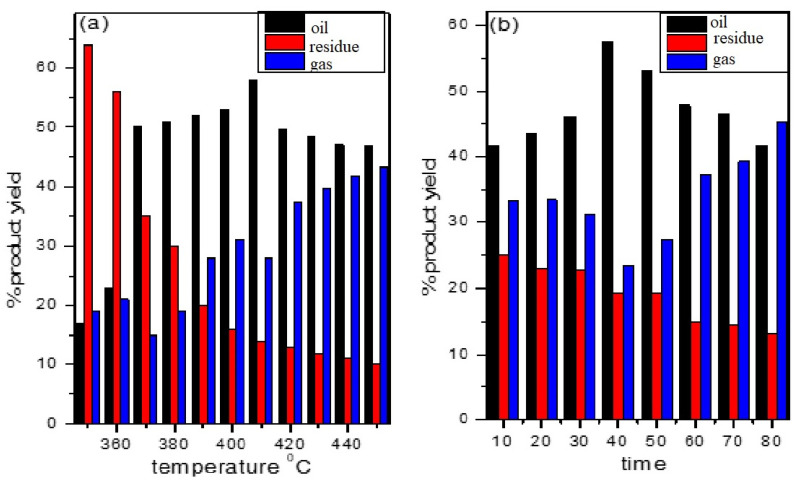
(**a**) Effect of temperature on pyrolysis products; (**b**) effect of time on product yield.

In a laboratory-scale pyrolysis setup, polypropylene waste was decomposed without the catalyst at various temperatures from 350 to 430 °C for 1 h; however, no oil was produced. Therefore, further experiments were performed using florisil as the catalyst, and a better yield of the liquid fraction was obtained. The optimum temperature for maximum oil production was determined by varying the temperature by 10 °C between 350 °C and 450 °C. The amount of the liquid fraction was observed to increase with the rise in temperature, from 17% at 350 °C to a maximum of 58% at 410 °C, and then began to decrease when the temperature exceeded 410 °C, as shown in Figure 4a. The amount of the gaseous fraction was generally observed to increase, and the solid residue decreased with the increase in temperature [30]. Many other researchers have reported similar findings, which are thought to be due to the cracking of C-C bonds at high temperature, resulting in lighter hydrocarbons with relatively short carbon chains [31,32]. Inguanzo et al. [33] noted that the increase in temperature caused a reduction in the solid fraction, while the increase in the gas fraction and the liquid fraction remained relatively constant. Similar findings were also reported by Williams et al. [34]. The authors speculated that the breakdown of the liquid products at elevated temperature resulted in an enhanced gas evaluation. Papuga et al. [35] explained the fact that at a higher temperature, secondary reactions begin to dominate, resulting in additional breaking of molecular chains, and hence the shorter chains arise during the process, resulting in non-condensable gases. Following the conclusion of the temperature optimization, further pyrolysis experiments were performed at intervals of 10 min from 10 to 80 min to establish the suitable time duration for the highest yield. The highest yield of oil, i.e., 57.41, was achieved in 40 min, as shown in Figure 4b. Hence, the optimum temperature and time for PP pyrolysis with florisil were observed to be 410 °C and 40 min, respectively. These results are somewhat consistent with previous findings [36]. Khan et al. [37] investigated the decomposition of model polypropylene over a molecular sieve at 350–390 °C and obtained the maximum yield of oil (42.5%) at 370 °C at a time duration of 60 min.

### 2.4. GC-MS

**Figure 5:**
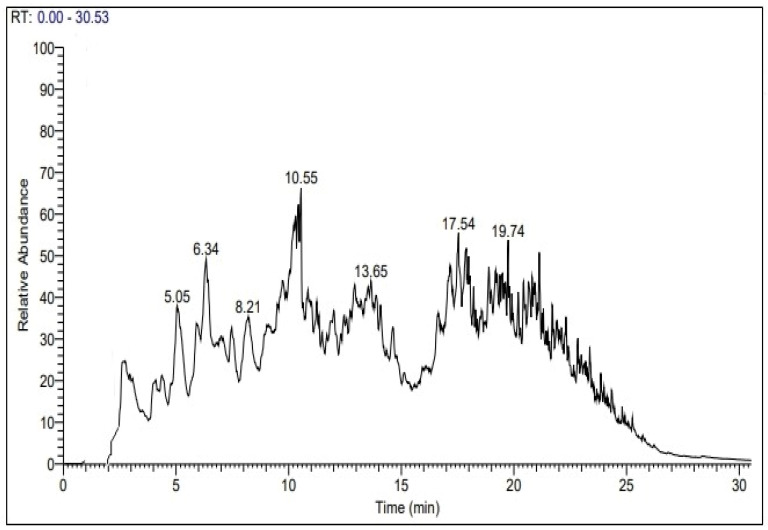
GC–MS of oil produced from PP waste degradation using florisil as the catalyst.

GC-MS was carried out to study the chemical structure and nature of the oil generated as a result of PP pyrolysis in the presence of florisil, and the obtained chromatogram is displayed in Figure 5, illustrating the detected compounds of the oil. Peaks corresponding to 2,4-Dimethyl-1-heptene (C_9_H_18_), Decane, 4-methyl- (C_11_H_24_), 3-Decene, 2,2-dimethyl-, (E) (C_12_H_24_), Cyclooctane, 1,4-dimethyl-, cis- (C_10_H_20_), 1,1,6,6-Tetramethylspiro [4.4]nonane (C_13_H_24_), 1-Nonadecene (C_19_H_38_), 17-Pentatriacontene (C_35_H_70_), Cyclohexane, 1,3,5-trimethyl-2-octadecyl (C_27_H_54_), Cyclotetradecane, 1,7,11-trimethyl-4-(1-methylethyl) (C_20_H_40_) and Dodecane, 1-cyclopentyl-4-(3-cyclopentylpropyl) (C_25_H_48_) were observed, which impart fuel characteristics to the pyrolysis oil. The data obtained are in agreement with some reported works. Shindikar et al. [38] characterized the oil generated by the thermal and catalytic pyrolysis of PP by GC-MS. The resulting chromatogram showed the existence of a petroleum fraction along with some high-molecular-weight hydrocarbons (C_20_–C_30_). Similarly, Nisar et al. [18] studied the decomposition of PP over a zeolite and found hydrocarbons in the range of C_4_–C_18_. Moreover, the oil was found to contain cyclic, aliphatic and branched-chain hydrocarbons.

### 2.5. FTIR

**Figure 6:**
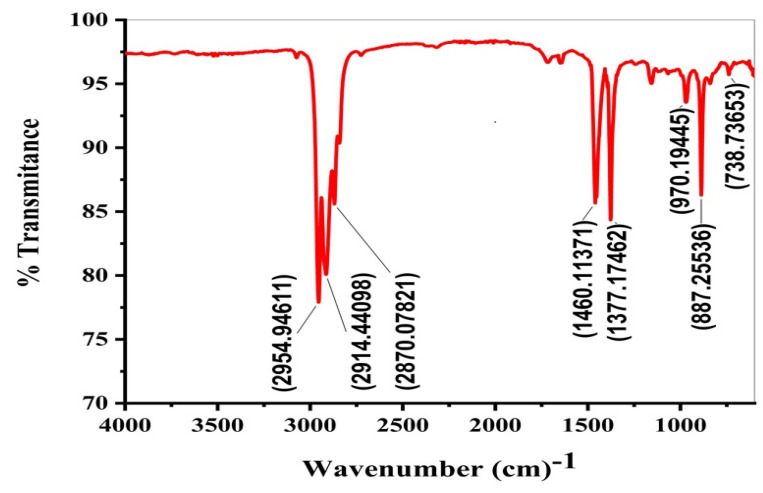
FTIR of oil recovered from the degradation of polypropylene waste.

Figure 6 shows the characteristic FTIR peaks for pyrolyzed PP oil. The peaks at around 2954 cm^−1^ and 2870 cm^−1^ relate to the symmetric and asymmetric stretching of CH_3_. The band at 2914 cm^−1^ was attributed to the asymmetric stretching of CH_2_**.** The peaks at 1460 and 1377 cm^−1^ indicate the symmetrical bending of CH_3_. The corresponding out-of-plane bending modes of C–H are depicted by the peaks at 887 cm^−1^. Additionally, the peaks at wave numbers of 970 cm^−1^ and 738 cm^−1^ correspond to the bending vibration of the C-H of alkene and alkane, and the phenyl ring substitution, respectively. Panda and Singh [39] derived oil from waste PP using kaolin clay at 400–550 °C and used FTIR analysis to evaluate the functional group content of the oil. They found many distinct peaks. The C-H stretching shown by the peaks at 2956 cm^−1^ and 2879 cm^−1^, the bending of alkane at 1377 cm^−1^ and the peak at 1456 cm^−1^ were attributed to the C-H stretching of alkene, whereas the peaks at 970 cm^−1^ were assigned to the C-H bending of alkene. These findings are consistent with our results to a great extent.

### 2.6. Fuel Properties

Various physicochemical parameters of the oil recovered from the decomposition of PP were determined and matched with the properties of commercial fuel, and the data are summarized in Table 2 [40,41]. The results show the similarity of the specific gravity and density to those of kerosene, while demonstrating the resemblance of the fluidity and kinematic viscosity to those of gasoline. The API gravity of the pyrolysis oil lies between the standard values of kerosene and gasoline. Viscosity is the only property that falls in the range of diesel. Hence, it can be concluded that the pyrolysis oil obtained from the decomposition of polypropylene waste is a blend of fossil fuels and therefore needs proper distillation for the recovery of individual fractions. The results are consistent with previous studies. Panda and Singh [39] estimated the specific gravity, density and viscosity of PP oil to be 0.7777, 0.7771 and 2.27, which agree well with our results. In another study, Ahmed et al. [42] produced oil from PP, compared its fuel properties with ASTM standards and found that the fuel properties of the oil were in accordance with the fuel grade requirement. 

## 3. Material and Methods

### 3.1. Material

Polypropylene waste was collected from a dump site in Peshawar city. Florisil in powder form (60–100 mesh) was obtained from BDH Chemicals Ltd., Poole, UK. Acetone (99.5% pure) was purchased from Sigma Aldrich, Saint Louis, MO, USA. The condenser, reaction vessel and connecting pipes were all made of Pyrex glass and were frequently cleaned with acetone after each pyrolysis experiment.

### 3.2. Thermogravimetric Analysis and Kinetic Study

Polypropylene was decomposed in an inert gas at 20 mL·min^−1^ in a thermogravimetric analyzer (TGA Q 500 TA, New Castle, DE, USA) in the presence of 5% florisil at temperature program rates of 3, 12, 20 and 30 °C·min^−1^ from 25 to 600 °C. The resultant data were utilized to derive the kinetic parameters using the following methods.

#### 3.2.1. Ozawa–Flynn–Wall

This is a good method for finding out kinetic parameters at different fraction conversions. The equation in its final form is

(1) $$\ln\beta =\ln\frac{\left[A.\mathrm{Ea}\right]}{\mathrm{Rg}\left(\alpha \right)}-5.3305-1.052\left(\frac{\mathrm{Ea}}{\mathrm{RT}}\right)$$

where β, Ea, A, T and R symbolize the heating rate, activation energy, pre-exponential factor, absolute temperature and universal gas constant, respectively. By plotting lnβ versus 1/T, the activation energy and frequency factor can be computed using the values of the slope and intercept, respectively.

#### 3.2.2. Kissinger–Akahira–Sunnose

The Kissinger–Akahira–Sunnose method presented in Equation (2) is considered as one of the reliable methods applied to thermogravimetric analysis to calculate kinetic parameters:

(2) $$\ln\left(\frac{\beta }{T^{2}}\right)=\;\ln\left[\frac{\mathrm{AR}}{\mathrm{Eag}\left(a\right)}\right]-\frac{\mathrm{Ea}}{\mathrm{RT}}$$

where β represents the heating rate, Ea is the activation energy, A is the pre-exponential factor, R is the universal gas constant and g(α) is the mathematical function for the reaction mechanism. By plotting $\ln\left(\frac{\beta }{T^{2}}\right)$ on the *y*-axis and $\frac{1}{T}$ on the *x*-axis, the activation energy and A-factor can be determined using the values of the slope and intercept.

### 3.3. Pyrolysis

**Figure 7:**
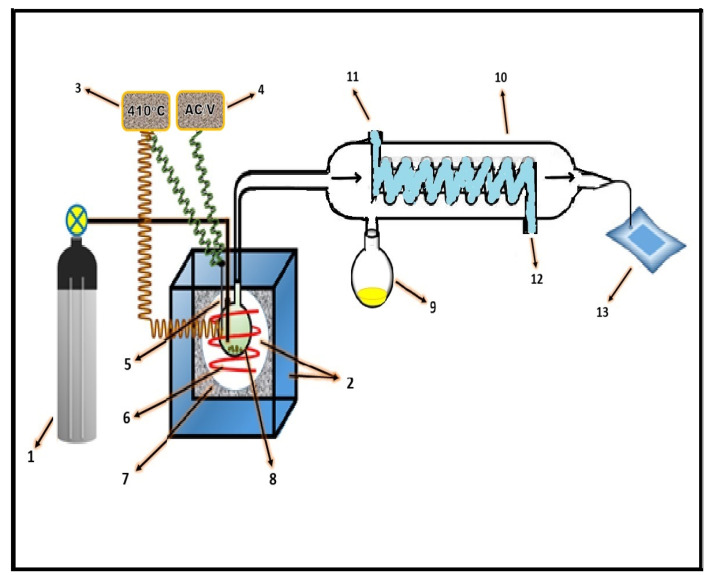
Experimental setup for pyrolysis: 1. nitrogen cylinder; 2. salt bath; 3. temperature controller; 4. power supply; 5. thermocouple; 6. coiled heater; 7. insulating material; 8. reaction vessel; 9. oil collection; 10. condenser; 11. water inflow; 12. water outflow; 13. gas-collecting bag.

Pyrolysis of waste polypropylene (≈5 g in each run) along with 5% catalyst was conducted in an indigenously made furnace (salt bath), as shown in Figure 7. The furnace consisted of a circular steel vessel with a diameter of 1 foot, enclosed in a 2-square-feet steel vessel, with insulating material between them. The circular vessel was filled with a mixture of salts and heated by a cable heater around the wall of the bath, having the capacity to retain a temperature of ≈480 °C. A thermocouple was used for sensing the temperature which was connected to a digital temperature controller for measuring and controlling the temperature. The sample was decomposed in a glass vessel hanging inside the molten salt. A constant flow of nitrogen was provided to keep the reaction atmosphere inert. The reaction vessel was connected to a condenser that was linked to a flask placed in an ice-cold bath for oil collection. The collected oil was analyzed by applying the GC-MS technique. The GC-MS instrument (Thermo scientific DSQ II) was equipped with a TR-5MS column (length 30 m, film thickness 0.25 μm, internal diameter 0.25 mm). Helium was used as a carrier gas with a flow rate of 1 mL·min^−1^. The sample was introduced into the GC with an initial oven temperature of 50 °C and held for 1 min, with the temperature then increased to 150 °C by 15 °C·min^−1^ with a hold time of 1 min and further increased to 280 °C by 6 °C·min^−1^. The NIST MS library was used for the identification of peaks. The oil was also studied using an FTIR spectrometer (Prestige-21, Shimadzu IR). The spectra obtained were compared with the available literature.

## 4. Conclusions

Thermo-catalytic degradation of polypropylene was performed over the temperature range of 350 to 450 °C in the presence of florisil. The temperature and reaction time were optimized for the maximum oil yield. The best parameters for the highest oil yield (57.41%) were found to be 410 °C and 40 min. The catalyst significantly lowered the activation energy as well as the degradation temperature. The oil produced was characterized using GC-MS and FTIR, which showed that the oil contained a wide range of hydrocarbons. The fuel characteristics of the oil were assessed using standard techniques, and the oil was found to be comparable with commercial fuel. This study will help in the degradation of polypropylene waste on a pilot scale.

## Figures and Tables

**Table 1:** Calculated kinetic parameters for PP degradation by the OFW and KAS equations.

α	Ozawa–Flynn–Waal Model						Kissinger–Akahira–Sunnose Model					
	Non-Catalytic			Catalytic			Non-Catalytic			Catalytic		
	Ea (kJ·mol^−1^)	A (min^−1^)	R^2^	Ea (kJ·mol^−1^)	A (min^−1^)	R^2^	Ea (kJ·mol^−1^)	A (min^−1^)	R^2^	Ea (kJ·mol^−1^)	A (min^−1^)	R^2^
0.1	109.95	9.1 × 10^8^	0.992	102.74	7.1 × 10^8^	0.994	108.76	4.0 × 10^8^	0.983	99.77	1.0 × 10^8^	0.992
0.2	119.43	6.3 × 10^9^	0.991	110.64	1.2 × 10^9^	0.99	113.08	7.3 × 10^8^	0.983	108.08	1.9 × 10^8^	0.992
0.3	127.46	9.3 × 10^9^	0.990	118.55	1.3 × 10^9^	0.992	121.91	9.1 × 10^8^	0.993	113.90	3.3 × 10^8^	0.993
0.4	141.52	9.9 × 10^10^	0.991	134.35	1.9 × 10^10^	0.993	129.70	9.9 × 10^9^	0.996	124.71	9.9 × 10^8^	0.996
0.5	151.58	7.9 × 10^11^	0.993	150.16	2.9 × 10^11^	0.997	139.38	3.0 × 10^10^	0.996	141.34	3.0 × 10^9^	0.996
0.6	167.61	9.1 × 10^11^	0.991	158.06	6.1 × 10^11^	0.999	153.32	6.5 × 10^11^	0.993	149.65	6.5 × 10^10^	0.999
0.7	174.83	1.2 × 10^12^	0.991	164.38	1.2 × 10^12^	0.999	168.16	1.9 × 10^12^	0.995	157.97	1.9 × 10^11^	0.995
0.8	183.57	6.3 × 10^12^	0.984	170.71	1.3 × 10^12^	0.984	173.13	7.1 × 10^12^	0.997	162.12	3.1 × 10^11^	0.999
0.9	198.66	9.3 × 10^12^	0.989	173.08	9.3 × 10^11^	0.989	184.28	9.3 × 10^12^	0.991	166.28	5.3 × 10^11^	0.991

**Table 2:** Comparison of the fuel properties of the oil obtained from PP waste with ASTM standard values.

S. No.	Parameters	This Work	ASTM Standard Values [40,41]		
			Diesel	Kerosene	Gasoline
1	Density (g/mL)	0.781	0.83–0.85	0.78–0.82	0.720–0.736
2	Fluidity	0.819	2.4–5.3	1.54–2.20	0.775–0.839
3	Viscosity (cP)	1.133	0.9–1.5	0.775–0.839	1.2–1.8
4	Specific gravity	0.743	0.83–0.85	0.72–0.73	0.78–0.82
5	API gravity	55.877	38.98–34.97	62.34–65.03	49.91–41.06
6	Kinematic viscosity (nm^2^/s)	1.591	1.3–5.3	1.076–1.140	1.54–2.20

## Data Availability

The data presented in this study are available on request from the corresponding author.
